# Not taught in medical school but needed for the clinical job – leadership, communication and career management skills for final year medical students

**DOI:** 10.1186/s12909-024-06091-w

**Published:** 2024-10-11

**Authors:** Felix Behling, Sasan Darius Adib, Patrick Haas, Hannes Becker, Linda Oberle, Eliane Weinbrenner, Isabella Nasi-Kordhishti, Constantin Roder, Jan Griewatz, Marcos Tatagiba

**Affiliations:** 1 Department of Neurosurgery and Neurotechnology, University Hospital Tübingen, Eberhard Karls University Tübingen, Hoppe-Seyler Street 3, Tübingen, Germany; 2 Hertie Institute for Clinical Brain Research, Tübingen, Germany; 3 Center for CNS Tumors, Comprehensive Cancer Center Tübingen-Stuttgart, University Hospital Tübingen, Eberhard-Karls University Tübingen, Tübingen, Germany; 4 Department of Neurology and Interdisciplinary Neuro-Oncology, University Hospital Tübingen, Eberhard Karls University Tübingen, Tübingen, Germany; 5 Department of Diagnostic and Interventional Neuroradiology, University Hospital Tübingen, Eberhard Karls University Tübingen, Tübingen, Germany; 6 Tübingen Institute for Medical Education (TIME), Eberhard Karls University Tübingen, Tübingen, Germany

**Keywords:** Final year medical students, Communication, Leadership, Career management, Residency

## Abstract

**Background:**

Starting the first job as a young physician is a demanding challenge. Certain skills are important to master this transformation that go beyond the theoretical knowledge and practical skills taught in medical school. Competencies such as communication, leadership and career management skills are important to develop as a young physician but are usually not sufficiently taught in medical school in a structured and comprehensive way.

**Methods:**

We performed an online survey among final year medical students regarding how they perceive their current competency level in communication, leadership and career management skills. We also assessed how they rate the importance to acquire these competencies and the current emphasis during their medical school education regarding these topics.

**Results:**

Of 450 final year medical students 80 took part in the voluntary survey and 75 complete datasets were returned (16.7%). The majority of respondents rated different communication skills, leadership skills and career management skills as important or very important for their later clinical work. However, most students felt to be poorly or very poorly prepared by the current medical school curriculum, especially for certain leadership and career management skills. Overall, 90.7% of participants expressed interest in an additional educational course that covers subjects of communication, leadership and career management skills during the later stage of medical school, preferably as a hybrid in-person session that also offers synchronous online participation.

**Conclusions:**

The results of the survey express the need to address communication, leadership and career management skills in the medical curriculum to be better prepare students for the demands of residency and their further course as physicians. An educational format during the final year of medical school may be suitable to address mentioned topics in the framework of clinical practical exposure.

**Supplementary Information:**

The online version contains supplementary material available at 10.1186/s12909-024-06091-w.

## Background

For final year medical students the start of their residency is a big transition, associated with feelings of doubt and uncertainty [1]. In Germany, the final year of medical school is designed to provide a continuous exposure to practical clinical work and preparation for the demands and challenges of the upcoming residency. Transferring theoretical knowledge into practice and becoming more familiar with the challenges of the clinical work is the main focus for final year medical students at this stage of their education [2].

However, with the start of residency young physicians will be faced with additional challenges that are usually not systematically taught in German medical schools but are requirements for a successful residency. One example is the importance of good communication skills. While communication with patients, such as breaking bad news, is an established part of the curriculum in some institutions [3], a multifaceted teaching of communication skills that includes interprofessional communication or conflict management is usually not included. However, it is an established fact, that most medical errors are based on miscommunication [4], underlining the need for medical students to be well educated in this regard.

Additionally, young physicians are placed into leadership settings from day one, usually with no prior training. The importance of leadership skills has been recognized in other countries and first concepts to address the educational need for this skill have been suggested [5–7]. For example, structured interviews among Canadian medical students provided data on the potential use of the 3-C model that focuses on character, competence and commitment as a framework for leadership development [5]. In the United Kingdom the Academy of Medical Royal Colleges and the NHS Institute for Innovation and Improvement have developed a Medical Leadership Competency Framework resulting in a Medical Leadership Curriculum offered to all medical specialties [6].

Additionally, young residents need to plan their individual career, regarding subspecialty training, additional qualifications or academic aspirations. These challenges add to the high demands along the way of becoming a board-certified physician and several publications support the positive impact of mentoring programs that facilitate individual professional developments [8, 9].

It is usually expected that students acquire all these skills on the fly during clinical rotations and residency. In the German medical school curriculum a structured teaching approach that comprehensively covers these skills has not yet been formulated [10]. This appears to be inappropriate when realizing the high importance of these competencies for the development as a competent physician.

Therefore, a structured analysis of final year medical students who are facing the transition from medical school to residency seems helpful to address this issue.

## Materials and methods

A web-based survey was constructed using the online platform “SurveyMonkey” (SurveyMonkey Inc., San Mateo, California, USA). The survey consisted of 18 questions evaluating the current status of the preparedness for residency, the integration of communication, leadership and career management skills into medical school education, as well as the interest to develop these skills and the wish to participate in a new education course during medical school that covers these subjects. The list of communication (*n* = 4), leadership (*n* = 5) and career management skills (*n* = 5) was assembled after focus group interviews with five former final year medical students who took part in a mentoring program in the first authors’ department during their final year of medical school [11] and have already made the transition to residency (orthopedics, psychiatry, anesthesia, internal medicine and urology) and experienced the associated challenges and demands.

For most questions a five-tiered Likert scale was used for expressing agreement with different statements or interest for specific teaching subjects. The complete survey is provided as supplementary material (Supplementary Material 1).

For the validation process the preliminary survey questions were sent to former final year medical students who were interviewed for collecting the topics of interest. Additional feedback was collected from the local Institute for Medical Education regarding improved phrasing and question order. Some active final year medical students in the authors’ department were asked to go through the questions and provide feedback on potential redundant or unspecific questions as a pilot cohort before the final survey was used.

The survey link was distributed to all medical students at the authors’ institution who were in their final year of medical school or preparing for the final oral exam via their institutional email addresses (*n* = 450). The survey was open from 30th March 2023 until 3rd of June 2023. Overall, 80 students participated in the survey producing 75 complete datasets (16.7%). The internal reliability of all Likert-scaled items was acceptable with a Cronbach’s alpha of 0.7618.

Approval by the ethical committee was not necessary since the survey was anonymous and completely voluntary.

Data processing was done with the JMP^®^ Statistical Discovery Software (Version 15.1.0, SAS Institute, Cary, North Carolina, US) and Microsoft^®^ Excel (Version 16.66.1, Microsoft, Redmond, Washington, US).

## Results

### Return rate and descriptive data of respondents

**Fig. 1:**
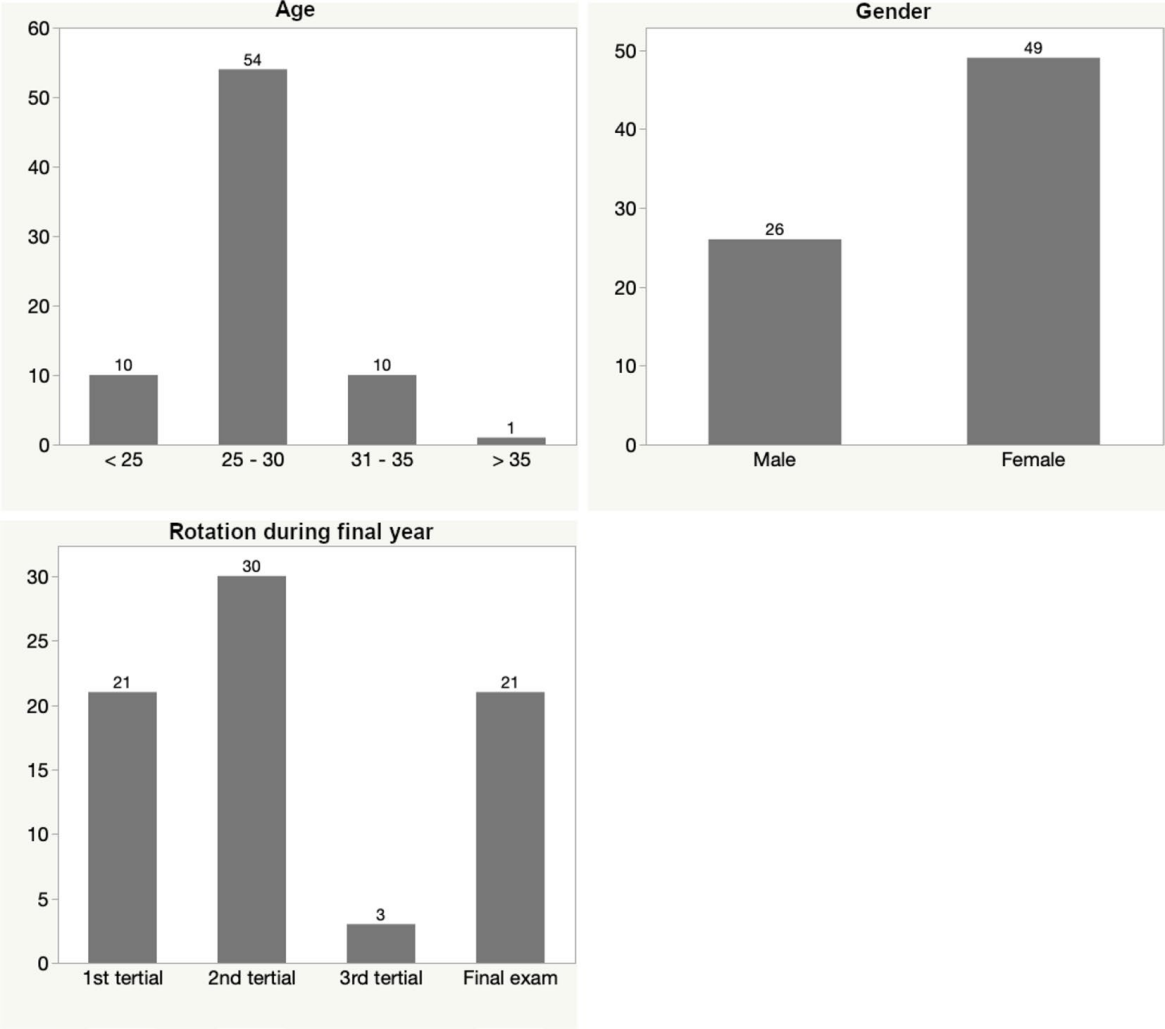
Bar graphs showing the distribution of participants’ age (**A**), gender (**B**) and level of final year training (**C**)

Overall, 80 of 450 final year medical students took part in the online survey (17.8%) and 75 complete datasets were submitted (16.7%), resulting in a completion rate of 93.8%. Forty-nine respondents were female (65.3%) and 26 were male with no participants indicating a diverse gender. The majority was 25 to 30 years of age (72.0%). Regarding the stage of the final year rotations, 21 respondents were in their first of three rotation (28.0%), while 30 were in their second and three in their third and final rotation (40.0 and 4.0%, respectively). Twenty-one students had just finished their three final year rotations and were preparing for the final oral exam (28.0%, see Fig. 1).

### Perceived importance of communication, leadership and career management skills

**Fig. 2:**
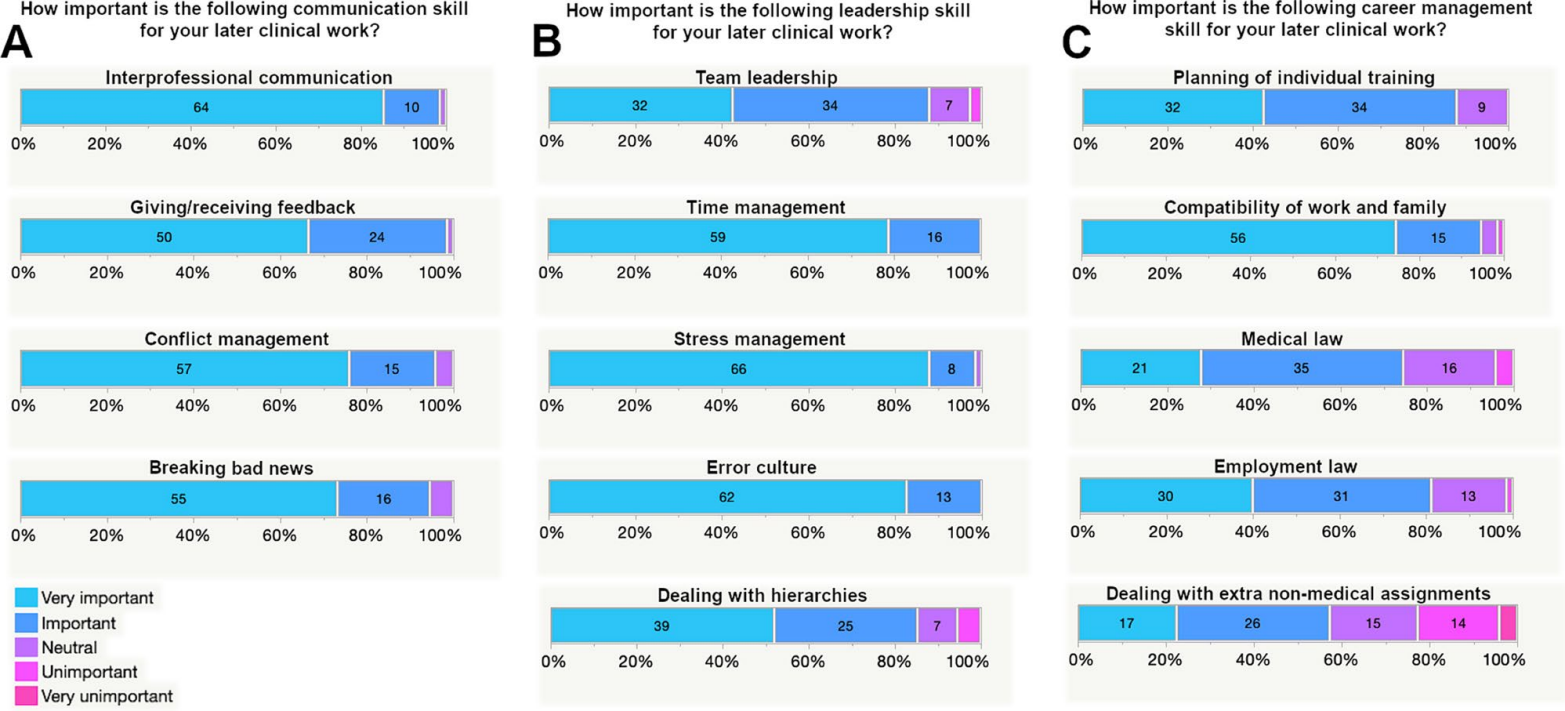
Distribution of participants’ answers regarding the perceived importance of different communication skills (**A**), leadership skills (**B**) and career management skills (**C**) for their later clinical career

When asked to rate the importance of different communication skills for their later clinical work, interprofessional communication was valued to be very important or important by 74 of 75 respondents (98.7%). The same result was seen regarding the importance of giving and receiving feedback (98.7% rated as very important or important). The management of conflicts was also assessed as very important or important by a large portion of respondents (72/75 respondents, 96.0%), so was the skill of breaking bad news (71/75 respondents, 94.7%). There were only a few deviating neutral responses and there were no participants who rated the different communication skills as unimportant or very unimportant (see Fig. 2A).

Among leadership skills, time management and error culture were rated as very important or important for the future clinical work by all respondents (100%). A similar result was observed for the subject stress management (98.7%) with only one neutral response. Team leadership was ranked as very important or important by 88.0% and dealing with hierarchies by 85.3% (see Fig. 2B).

The importance of different career management skills was confirmed by the majority of participants. Planning of individual training reached the highest agreement regarding its importance with 66 of 75 respondents (88.0%) and 9 neutral answers (12.0%). Compatibility of work and family was valued as important or very important by 94.7% with 3 neutral responses and one individual rating the subject as unimportant for later clinical work. A large portion of students agreed to the importance of medical law and employment law for their future career (74.7 and 81.3%, respectively). Few respondents felt that dealing with additional non-medical assignments was of no importance (22.7%) or gave a neutral response (20.0%), while the majority rated this career management skill as important or very important (57.3%, see Fig. 2C).

### Current curricular preparation for communication, leadership and career management skills

**Fig. 3:**
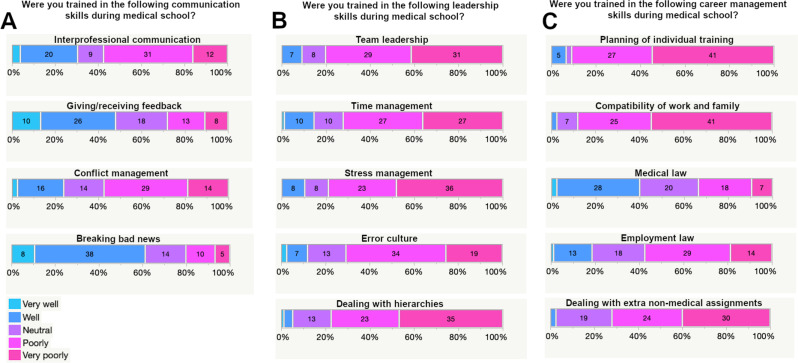
Distribution of participants’ answers regarding the current training for communication skills (**A**) leadership skills (**B**) and career management skills (**C**) during medical school

When asked about their level of preparedness in different aspects of communication, differing responses were given: A large group of respondents felt well or very well trained to break bad news to patients (46/75, 61.3%) and only a small portion felt poorly or very poorly prepared (20.0%). Regarding the skill of giving and receiving feedback a similar result was seen, with half of the participants stating to be well or very well prepared (36/75, 48.0%) and a quarter to be poorly or very poorly trained (21/75, 28.0%). When asking about preparation in interprofessional communication the majority of respondents stated to be poorly or very poorly trained (43/75, 57.3%). The subgroup of participants who answered to be poorly or very poorly trained for conflict management was of the same size (43/75, 57.3%) (see Fig. 3A).

The level of preparation during medical school for all addressed subjects of leadership was rated mostly negative. 80% felt poorly or very poorly prepared for team leadership (60/75), 72.0% for time management (54/75), 78.7% for stress management (59/75), 70.7% for error culture (53/75) and 77.3% for dealing with hierarchies (58/75) (see Fig. 3B).

The conceived preparation regarding career management skills showed a high rate of poor training as well. The preparation for planning of individual training is felt to be poor or very poor by 90.7% (68/75) and 88.0% for compatibility of work and family (66/75). Dealing with extra non-medical assignments is also felt to be addressed poorly or very poorly during medical education (54/75, 72.0%). In contrast the majority of respondents feel well or very well prepared in the field of medical law (30/75, 40.0%) but poorly or very poorly in employment law (43/75, 57.3%). For both legal subjects, quite a large group gave a neutral response, 26.7% for medical law and 24.0% for employment law (see Fig. 3C).

### Recommendations for educational formats

When asked about possible educational formats to address the evaluated contents of communication, leadership and career management skills, sixty-eight respondents expressed an interest (90.7%). Three gave a neutral response (4.0%) and four had no interest (5.3%). The majority of participating students responded that the second half of their clinical education or the final year of medical school would be best suited (73.3 and 77.3%, respectively) and one or two hours per week was the most desired timely scope (34.7 and 25.3%, respectively), followed by one hour every other week (22.7%). Respondents preferred an interactive seminar and group discussions (88.0 and 72.0%, respectively). Simulations with patient actors and role playing were favored by 41.3 and 38.7%, respectively. Frontal lecture and group work were the least desired teaching formats (28.0 and 24.0%, respectively). Only 10 respondents were in favor of a synchronous webinar as course format (13.3%) and 27 preferred an in-person session (36.0%), while the majority voted for a hybrid in-person session that also offers synchronous online participation (50.7%).

## Discussion

The results of the survey show a clear demand of final year medical students to receive specific and structured education on several topics of communication, leadership and career development skills which are usually not comprehensively taught in German medical schools.

### Communication skills

There have been some institutions in Germany and in other countries that address certain aspects of communication skills, such as breaking bad news. A widely used tool is the integration of patient actors for training scenarios where medical students experience the demands and challenges of a staged difficult communication setting, with prior preparation and later discussion and feedback [12, 13]. A similar approach has been established for several years in the authors’ institution, explaining that over 60% of the respondents felt well or very well prepared to deliver bad news to patients. Learning from this conceptual example, many other difficult scenarios could potentially be taught in this manner, such as interprofessional conflict management or giving and receiving feedback, just to name a few. As an example, it has recently been shown that a realistic learning activity on interprofessional communication can be created, combining students from different professions (nursing, physiotherapy, occupational therapy and physician) [14], underlining the feasibility of interprofessional teaching concepts.

### Leadership skills

Participants rated all listed leadership skills as important or very important for their later clinical job but expressed poor or very poor preparation within their current medical training.

In the corporate world it is long established that personnel with aspirations after leadership positions undergo proper and structured training to meet the demands of the job. It is incomprehensible that this is not the case for medical students who will enter their residency unprepared for leadership roles, with patient safety and lifes at stake [15]. The need to address this discrepancy is reflected in the quantity of publications on this matter. In a recent review the current standing of leadership training models for medical students are discussed, focusing on the diversity of methodology and outcome measurements. It becomes clear that courses addressing leadership skills at different stages of training during medical school are well accepted but it is still challenging to produce objective long-term outcome data and assess the longitudinal benefit for participants [16]. A structured leadership training program recently demonstrated that a longitudinal assessment is feasible and produces feedback for further improvement [17].

Comparing the growing evidence on the importance of leadership training for young physicians and the perceived lack of it in the German medical school curriculum, a clear mission to provide a structured and comprehensive curriculum including leadership skill training can be formulated.

### Career management skills

How young physicians see their future career and what they plan to accomplish is highly variable. While all residents who enter a certain residency share a similar goal of being well trained, interests for sub-specialization or further qualifications, as well as higher academic aspirations are highly individual. Of course a career is influenced by personal life events and change of career plans that are naturally not always predictable. Nonetheless, it is important for young residents to be cognizant of early planning and structuring of one’s individual career goals. Many medical schools offer mentoring programs, which often includes career counseling and professional development [18, 19]. Its impact on residency training has also been shown [20]. A career counseling program was also tested in Germany 10 years ago and documented good acceptance among medical students. 12% of participants changed their career choice after the program [21]. It must be stressed that career management skills are not just a nice thing to have, but the lack of it is a source of stress among medical students and young residents. A survey of over 900 medical students in Florida assessed perceived stressors. Students highlighted fear of excessive workload, time management, work-life balance as well health concerns and financial problems, but some students also indicated concerns about lack of assistance with career planning [22]. Furthermore, the new generation of medical students seems to have different career expectations and an increasing emphasis on work-life balance of as well as less eagerness to pursue an academic career [23–25]. Therefore, guidance in career management will remain an important aspect of medical student and young physician mentoring, with a broader diversity of individual career blueprints to consider. Mentoring in this regard should therefore not just focus on students with high potential and academic aspirations, but especially offer advice to the whole spectrum of medical students and trainees, which in turn requires more career diversity of potential mentors as well.

### Limitations

Although the results of the survey express the need to formulate a supportive educational concept, it is important to keep in mind, that the survey only delineates the current opinion of final year medical students in the authors’ institution that participated in a voluntary survey. The main limitation is the low response rate of 16.7%, which may express the overstraining of medical students by evaluations and surveys but may also reflect a low interest in the topic. It should be kept in mind that the voluntary design of the survey may overrepresent respondents that may have been more concerned and critical regarding their future. Additionally, the results of the survey may not be applicable to other institutions. However, due to the similar curricular presets in German medical faculties the main message is probably transferrable.

The items of the survey represent topics that young residents expressed as important during focused interviews. Respondents of the survey may lack the clinical exposure of residency to really understand the importance of some of the issues that they will face. However, it is our experience after having mentored many final year medical students that most are quite mindful of the challenges that they are about to face when commencing their residencies.

Additionally, the participating students were subject to the academic educational recession during the COVID-19 pandemic that highly influenced clinical practical education during medical school [26, 27]. This might have influenced the results of the survey by increased concern of respondents regarding their residency. Furthermore, the desired conditions of a new educational course, such as hybrid format, are important impulses, but may not be feasible or in line with the best suited didactic methodologies.

### A mandate for a supporting educational format

Participants provided data for a clear demand to formulate a comprehensive and structured curriculum that covers communication, leadership and career management skills. While single aspects may be already covered in some institutions, medical students do not feel sufficiently prepared in this regard to meet the demands of residency that they are soon to experience. Therefore, it is plausible to offer a respective educational format at the end for their training, as desired by most respondents of the survey. The clinical practical exposure during this time of training sets the perfect stage where such skills and competencies can be developed and applied. Furthermore, group discussions were chosen as the preferred format with an extent of 1 to 2 h every 1 or 2 weeks in a hybrid format. Although, the time frame may be sufficient, it remains questionable if a hybrid format holds the same educational yield as a seminar in attendance, especially when group discussions are implemented.

Based on the results of this survey, we are currently developing a teaching session for final year medical students. By covering the subjects that were rated as being of the highest importance we want to address the additional demands of future physicians. Furthermore, we plan to assess the development of course participants longitudinally with a focus on the usefulness of the contents of our teaching session regarding individual professional development during residency. This will give us the chance to regularly re-assess our teaching format and adjust it accordingly.

## Conclusions

Final year medical students in Germany express a lack of communication, leadership and career management skills in their current medical school curriculum, suggesting the introduction of a supportive educational format that addresses these topics to be better prepared for the challenges of residency.

## Electronic supplementary material

Below is the link to the electronic supplementary material.

Supplementary Material 1

Supplementary Material 2

## Data Availability

The dataset analyzed during the current study is available as supplementary file.
