# Supplementary material for: Not taught in medical school but needed for the clinical job – leadership, communication and career management skills for final year medical students
Source: BMC Med Educ. 2024 Oct 11;24:1126. doi: 10.1186/s12909-024-06091-w (PMC11468378; doi:10.1186/s12909-024-06091-w)
Supplement: Supplementary file 2 — Supplementary Material 2 [file 12909_2024_6091_MOESM2_ESM.pdf]

Leadership and Career Management Skills for upcoming physicians

Dear final-year medical students,

as you begin your residency, you will soon face challenging situations, conflicts, health policy and legal issues, as well as your own career planning. In addition to the specialist knowledge from approximately 30 medical fields, medical students are not specifically prepared for these demands during their studies. These topics are also not addressed within the framework of residency.

This survey aims to assess the current training situation regarding these topics. The survey is voluntary, anonymous, and takes 5 minutes to complete.

Thank you for your participation!

PD Dr. med. Felix Behling
Lehrbeauftrager oft he Deparment for Neurosurgery
Contact: felix.behling@med.uni-tuebingen.de

1. What rotation are you currently in?

- ○ 1st tertial
- ○ 2nd tertial
- ○ 3rd tertial
- ○ Final exam

2. **Do you feel sufficiently prepared regarding the following demands of your upcoming residency?**

	Strongly agree	Agree	Neutral	Disagree	Strongly disagree
Medical Knowledge	○	○	○	○	○
Practical skills	○	○	○	○	○
Communication skills	○	○	○	○	○
Team leadership	○	○	○	○	○
Conflict management	○	○	○	○	○

3. How important is the following communication skill for your later clinical work?

	Very important	Important	Neutral	Unimportant	Very unimportant
Interprofessional communication	○	○	○	○	○
Conflict management	○	○	○	○	○
Giving/receiving feedback	○	○	○	○	○
Breaking bad news	○	○	○	○	○

4. **How important is the following leadership skill for your later clinical work?**

	Very important	Important	Neutral	Unimportant	Very unimportant
Team leadership	○	○	○	○	○
Time management	○	○	○	○	○
Stress management	○	○	○	○	○
Error culture	○	○	○	○	○
Dealing with hierarchies	○	○	○	○	○

5. **How important is the following career management skill for your later clinical work?**

	Very important	Important	Neutral	Unimportant	Very unimportant
Planning of individual training	○	○	○	○	○
Compatibility of work and family	○	○	○	○	○
Medical law	○	○	○	○	○
Employment law	○	○	○	○	○
Dealing with extra non-medical assignments	○	○	○	○	○

6. Were you trained in the following communication skills during medical school?

	Very well	Well	Neutral	Poorly	Very poorly
Interprofessional communication	○	○	○	○	○
Conflict management	○	○	○	○	○
Giving/receiving feedback	○	○	○	○	○
Breaking bad news	○	○	○	○	○

7. Were you trained in the following leadership skills during medical school?

	Very well	Well	Neutral	Poorly	Very poorly
Team leadership	○	○	○	○	○
Time management	○	○	○	○	○
Stress management	○	○	○	○	○
Error culture	○	○	○	○	○
Dealing with hierarchies	○	○	○	○	○

8. **Were you trained in the following career management skills during medical school?**

	Very well	Well	Neutral	Poorly	Very poorly
Planning of individual training	○	○	○	○	○
Compatibility of work and family	○	○	○	○	○
Medical law	○	○	○	○	○
Employment law	○	○	○	○	○
Dealing with extra non-medical assignments	○	○	○	○	○

9. Would you have been interested in an accompanying course during your medical studies that covered the aforementioned subject areas?

- Very interested
- Interested
- Neutral
- Uninterested
- Very uninterested

10. Which soft skills topics should be included in such a course?

- Interprofessional communication
- Conflict management
- Giving/receiving feedback
- Breaking bad news
- None
- Other

---

11. Which topics on leadership skills should be included in such a course?

- Team leadership
- Time management
- Stress management
- Error culture
- Dealing with hierarchies
- None
- Other

____

12. Which topic areas on career management skills should be included in such a course?

- Planning of individual training
- Compatibility of work and family
- Employment law
- Medical law
- Dealing with extra non-medical assignments
- None
- Other

____

13. What time do you think is optimal for training in the topics mentioned (multiple answers possible)?

- Early clinical phase of medical school
- Later clinical phase of medicla school
- Final year of medical school
- With the beginning of residency
- During the course of residency
- Other

---

14. What length of time for a corresponding course do you consider to be optimal?

- 1 hour per week
- 2 hours per week
- 3 hours per week
- 1 hour every 2 weeks
- 2 hours every 2 weeks
- 3 hours every 2 weeks
- Other

---

15. Which educational format would you prefer?

- Synchronous webinar
- In-person session
- Hybrid format (in-person session and webinar)
- Other

---

16. Which teaching formats do you think are useful for teaching the topics mentioned (multiple answers possible)?

- ○ Frontal lecture
- ○ Interactive seminar
- ○ Role playing
- ○ Patient actors
- ○ Group discussions
- ○ Other

---

17. How old are you?

- ○ < 25
- ○ 25 – 30
- ○ 30 – 35
- ○ > 35

18. Gender

- Female
- Male
- Diverse

Thank you for your participation!

PD Dr. med. Felix Behling
Lehrbeauftrager oft he Deparment for Neurosurgery
Contact: felix.behling@med.uni-tuebingen.de
